# Functional genomic analysis of the isolated potential probiotic *Lactobacillus delbrueckii* subsp. *indicus* TY-11 and its comparison with other *Lactobacillus delbrueckii* strains

**DOI:** 10.1128/spectrum.03470-23

**Published:** 2024-05-21

**Authors:** Sk. Md. Jakaria Al-Mujahidy, Kirill Kryukov, Kazuho Ikeo, Kei Saito, Md. Ekhlas Uddin, Abu Ali Ibn Sina

**Affiliations:** 1 DNA Data Analysis Laboratory, Department of Genomics and Evolutionary Biology, National Institute of Genetics, Mishima, Shizuoka, Japan; 2 Center for Genome Informatics, Joint Support-Center for Data Science Research, Research Organization of Information and Systems, Mishima, Shizuoka, Japan; 3 Bioinformation and DDBJ Center, National Institute of Genetics, Mishima, Shizuoka, Japan; 4 Laboratory of Physics and Cell Biology, National Institute of Genetics, Mishima, Shizuoka, Japan; 5 Department of Biochemistry and Molecular Biology, Gono Bishwabidyalay, Savar, Dhaka, Bangladesh; 6 Australian Institute for Bioengineering & Nanotechnology (AIBN), The University of Queensland, Brisbane, Queensland, Australia; 7 Department of Systems Biology, Columbia University Irving Medical Center, New York, New York, USA; University of Minnesota Twin Cities, St. Paul, Minnesota, USA

**Keywords:** TEM, draft genome sequencing, the strain TY-11

## Abstract

Probiotics refer to living microorganisms that exert a variety of beneficial effects on human health. On the contrary, they also can cause infection, produce toxins within the body, and transfer antibiotic-resistant genes to the other microorganisms in the digestive tract necessitating a comprehensive safety assessment. This study aimed to conduct functional genomic analysis and some relevant biochemical tests to uncover the probiotic potentials of *Lactobacillus delbrueckii* subsp. *indicus* TY-11 isolated from native yogurt in Bangladesh. We also performed transmission electron microscopic (TEM) analysis, comparative genomic study as well as phylogenetic tree construction with 332 core genes from 262 genomes. The strain TY-11 was identified as *Lactobacillus delbrueckii* subsp. *indicus,* whose genome (1,916,674 bp) contained 1911 CDS, and no gene was identified for either antibiotic resistance or toxic metabolites. It carried genes for the degradation of toxic metabolites, treatment of lactose intolerance, toll-like receptor 2-dependent innate immune response, heat and cold shock, bile salts tolerance, and acidic pH tolerance. Genes were annotated for inhibiting pathogenic bacteria by inhibitory substances [bacteriocin: Helveticin-J (331 bp) and Enterolysin-A (275 bp), hydrogen peroxide, and acid]; blockage of adhesion sites; and competition for nutrients. The genes involved in its metabolic pathway were detected as suitable for digesting indigestible nutrients in the human gut. The TY-11 genome possessed an additional 37 core genes of subspecies *indicus* which were deficient in the core genome of the most popular subsp. *bulgaricus*. During the phenotypic testing, the isolate TY-11 demonstrated high antagonistic activity (inhibition zone of 21.33 ± 1.53 mm) against *Escherichia coli* ATCC 8739 and was not sensitive to any of the 10 tested antibiotics. This study was the first study to explore the molecular insights into probiotic roles, including antimicrobial activities and antibiotic sensitivity, of a representative strain (TY-11) of *Lactobacillus delbrueckii* subsp. *indicus*.

**IMPORTANCE:**

This study aimed to conduct functional genomic analysis to uncover the probiotic potential of *Lactobacillus delbrueckii* subsp. *indicus* TY-11 isolated from native yogurt in Bangladesh. We also performed transmission electron microscopic (TEM) analysis, comparative genomic study as well as phylogenetic tree construction with 332 core genes from 262 genomes. In our current investigation, we revealed a number of common and unique excellences of the probiotic *Lactobacillus delbrueckii* subsp. *indicus* TY-11 that are likely to be important to illustrate its intestinal residence and probiotic roles. This is the first study to explore the molecular insights into intestinal residence and probiotic roles, including antimicrobial activities and antibiotic sensitivity, of a representative strain (TY-11) of *Lactobacillus delbrueckii* subsp. *indicus*.

## INTRODUCTION

The species *Lactobacillus delbrueckii* consists at present of six subspecies, *delbrueckii, lactis, bulgaricus, indicus, sunkii,* and *jakobsenii* showing a high level of DNA-DNA hybridization similarity but presenting a few markedly different phenotypic and genotypic characters of different ecological adaptation and restricted number of carbohydrates. Subspecies *bulgaricus, indicus,* and *lactis*, which were first isolated from dairy-based products, are all lactose positive, whereas *delbrueckii, sunkii,* and *jakobsenii,* which were first isolated from non-dairy-based products, are lactose negative (1). Although *Lactobacillus delbrueckii* subsp. *bulgaricus* isolated mainly from fermented milk, it has recently also been detected in Bulgarian plants. Most isolates of the other recognized subspecies of *L. delbrueckii, Lactobacillus delbrueckii subsp. lactis* have come from cheeses. In 2004, four strains of *Lactobacillus delbrueckii* subsp. *sunkii*, designated YIT 11220, YIT 11,221T, YIT 11466, and YIT 11673, were isolated from samples of sunki, which is a traditional, Japanese, non-salted pickle that is produced by the fermentation of the leaves of red turnips (‘otaki-kabu’) (2). *Lactobacillus delbrueckii* subsp. *delbrueckii*, missing a lactose operon, was first observed in fermented plant extracts (3), while *Lactobacillus delbrueckii* subsp. *indicus*, which has lactose fermentation characteristics, had been isolated from fermented dairy products of India (4). The last discovered subspecies is *Lactobacillus delbrueckii* subsp. *jakobsenii*. A novel isolate, designated ZN7a-9T, was named *Lactobacillus delbrueckii* subsp. *jakobsenii* and isolated from malted sorghum wort used for making an alcoholic beverage (dolo) in Burkina Faso (1).

Interestingly, while subsp. *bulgaricus, indicus,* and *lactis* were all first isolated from dairy-based products and are all lactose positive; only two subspecies, *lactis* and *bulgaricus,* account for the commercial relevance. Subspecies *indicus* has not been marketed yet. The other three subspecies (*delbrueckii, sunkii,* and *jakobsenii*) also have no economic pertinence, notwithstanding they are important from an evolutionary perspective because they maintain distinct nutrition and habitats. The ongoing genome sequencing of these subspecies will also aid in the illustration of further properties that probably be of future financial relevance or provide new insights on the genetic information (1).

In our study, our goal was to isolate probiotic *Lactobacillus delbrueckii* subsp. *indicus* TY-11. The subspecies indicus was reported previously by two different studies, where phenotypic and genotypic traits were observed by DNA–DNA hybridization, multilocus sequence typing (MLST), 16S rRNA gene sequencing, and biochemical tests (4, 5). From the past two studies, it was confirmed that subspecies *indicus* strains were potential probiotics, which fermented lactose constitutively as subspecies bulgaricus. However, Dellaglio et al. (4) differentiated subspecies *indicus* from other five subspecies by PCR products of one or two genes, which are present in these five subspecies contrasting with subspecies *indicus*. On another point, prior studies did not focus on the responsible genes for the fermentation of lactose in subspecies *indicus*. Genome sequence and comparative genomic study can be used to explore the mysterious genes responsible for lactose fermentation. This study is also required for the proper identification and preparing a comprehensive phylogenetic tree including all six subspecies of *Lactobacillus delbrueckii*. Moreover, this genome sequence is useful to explore the genes of our isolated strain for inhabitation in the human intestine, robustness in the areas of mobile genetic elements (MGEs), adaptation to environmental stress conditions, metabolism of nutrients and toxic metabolites, recovering digestive disorders, secretion system, extracellular matrix, immunity, adhesion, flavor-producing capabilities, antimicrobial activities, and bacteriocin biosynthesis, which are important probiotic properties. In the previous studies, this information was not covered for the strains of subspecies *indicus*. Only one previous research covered some functional genes and comparative genomic information from the genome sequences of the *indicus* subspecies, but not enough (6). Therefore, this study aims to isolate and identify a potential strain of *L. delbrueckii* subsp. *indicus* from yogurt in Bangladesh and conduct genome sequencing to uncover its genomic attributes for the above-mentioned potential features. In addition, the cellular morphology was examined using a transmission electron microscope (TEM), and the genetic characteristics of the tested isolate were verified utilizing a few biochemical assays.

## RESULTS

### Isolation of bacterial samples

#### Isolation of bacterial samples based on morphology, acid secretion efficiency, and anti-*E*. *coli* activity

We selected 15 colonies on MRS (de Man Rogosa Sharpe, Oxoid, UK) agar medium from several yogurt samples in Tongi, Gazipur, Bangladesh. Based on the visual observation of morphology, several bacterial colonies have similarities with *L. delbrueckii* subsp. *indicus* were selected from MRS agar media. The colonies were large, glossy on top, irregular-edged, and opaque (5).

One of our long-term targets was to isolate a probiotic strain, which can be an affordable choice for treating human sickness caused by diarrhea and other food-related illnesses linked to harmful *E. coli*. While the primary aim of this investigation was to identify a probiotic strain of *L. delbrueckii* subsp. *indicus*, we also assessed the anti-*E*. *coli* activity of each isolate to connect this study to our long-term objective. Despite the fact that it was not possible to complete all the necessary experiments in the current study to establish our long-term goal, we performed antagonistic tests of our target potential probiotic strains against only *Escherichia coli* ATCC 8739 during primary selection. From the primary selection of 15 isolates, 60% of bacterial strains (nine strains) demonstrated excellent acid production capacity and anti-*E*. *coli* activity. The inhibition zone against *E. coli* and the acid production capacity of nine isolates have been cited in the table (Table 1). With a zone of inhibition (ZOI) of 21.33 ± 1.53 mm, the strain TY-11 demonstrated the strongest anti-*E*. *coli* activity, as can be seen from the table. From the viewpoint of morphology, acid secretion, and anti-*E*. *coli* activity, we selected three strains: TY-11, TY-3, and TB-3, which demonstrated the ZOI of 21.33 ± 1.53 mm, 21.0 ± 1.0 mm, and 17.17 ± 1.44 mm, respectively.

**TABLE 1:** The antagonistic test against *Escherichia coli* ATCC 8739 and acid production of nine isolates

Serial number	Isolate code	Diameter of ZOI (mm)	pH
1	TB-1	16.0 ± 0.0	4.23 ± 0.0
2	TB-2	15.0 ± 0.0	4.23 ± 0.0
3	TB-3	17.17 ± 1.44	4.26 ± 0.0
4	TY-2	13.0 ± 0.0	4.23 ± 0.32
5	TY-3	21.0 ± 1.0	4.04 ± 0.0
6	TY-6	19.0 ± 2.0	4.01 ± 0.19
7	TY-7	17.0 ± 4.58	4.18 ± 0.32
8	TY-11	21.33 ± 1.53	4.22 ± 0.19
9	TY-12	15.0 ± 0.0	4.44 ± 0.35

#### 16S sequencing and molecular identification of bacterial samples

Among three isolates (TY-11, TB-3, and TY-3), the isolate TY-11 was identified as *L. delbrueckii* subsp. *indicus*. The GenBank accession number of the isolate TY-11 was OQ652026. 16S ribosomal RNA of TY-11 showed 99.84% identity with the closest 16S ribosomal RNA of *L. delbrueckii* subsp. *indicus* strain NCC725 (subject and query sequence lengths were 1,515 bp and 1,283 bp, respectively).

#### Colony morphology of the isolate TY-11 under a stereo microscope

**Fig 1:**
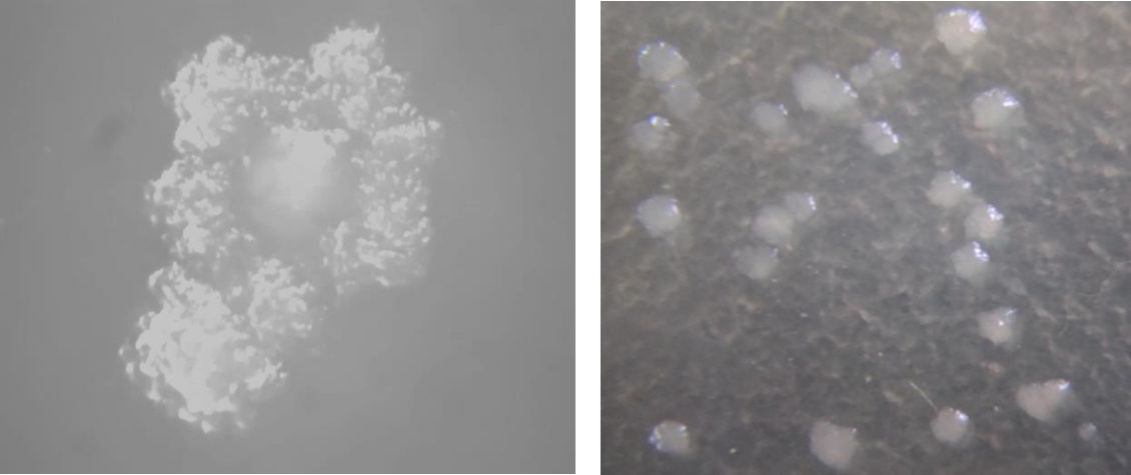
The colonies of the TY-11 were inspected under a stereo microscope, Leica MZ9.5 (Germany) with magnification levels up to 480×. The left image was taken at a larger magnification than the right image.

The colonies of TY-11 were large, opaque, white, umbonate (possessing knobby protuberances), and shiny in surface, as well as irregular in edge (Fig. 1). Only one study (5) described previously the colony morphology of *L. delbrueckii* subsp. *indicus* (5). Our observation was slightly different from Changjun et al. (5) because we examined the morphology with a different stereo microscope, Leica MZ9.5 (Germany) besides visual inspection.

#### Transmission electron microscope analysis of the isolate TY-11

**Fig 2:**
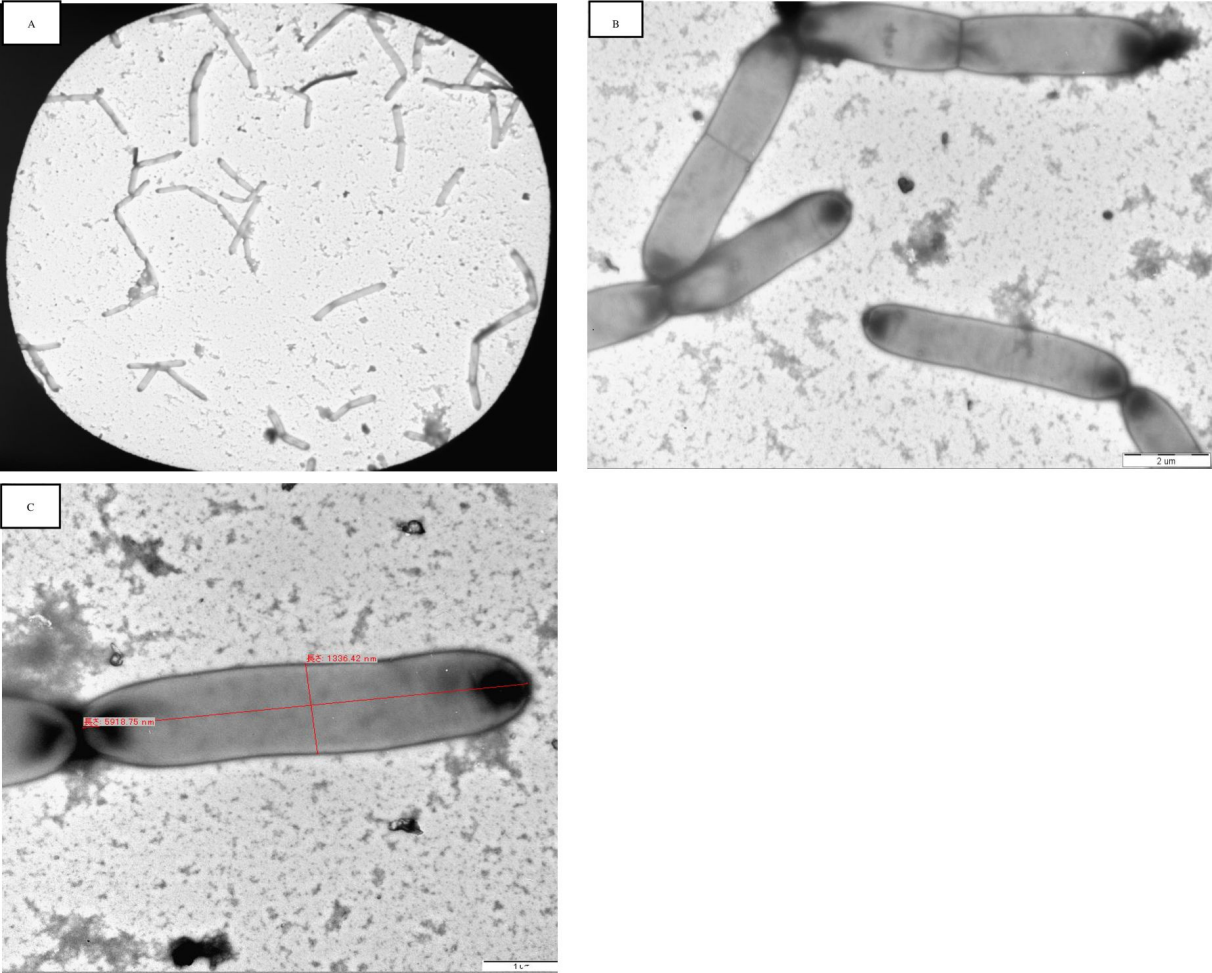
The figure highlights the *L. delbrueckii* subsp. *indicus* strain TY-11 cells’ microscopic images. The cells are shown in chains at a 1K and 10 K magnification in pictures (A) and (B). A cell of the strain TY-11 in photo (C) at 20 K magnification displays cell length = 5979.89 nm and width = 1337.42 nm.

After morphological and 16S sequencing result analysis, the strain TY-11 was selected for further studies. Rod-shaped cells of varying lengths and widths (2.2−9 µm lengths and 0.7−1.4 µm widths) with rounded ends were observed under TEM (Fig. 2). It is the first TEM analysis report for a strain of subsp. *indicus*.

### Genome sequencing of the isolate TY-11 and QC check of genome sequence

According to the FastQC report, the total number of reads was 4,135,806 (2,067,903 pairs), and their total size was 1.3 gigabases. The sequence length was 35−53 bp, and the %GC was 50. This sequence length varied from a shorter read to a longer read. According to the report of fastp (version 0.19.7), the mean length of paired-end raw data before filtering was 134 bp, 135 bp, and after filtering it was 131 bp, 131 bp. The duplication rate was 0.944674%, and the insert size was 35 bp.

### Genome composition analysis of the genome TY-11 by assembly and annotation

In the TY-11 genome, the total length of coding regions was 1,916,674 bases. The total length of coding regions accounted for 99.48% of the draft genome, whereas noncoding regions were comprised of only 0.52% nucleotides of the draft genome. The genome had 122 contigs, 69 tRNA, 4 rRNA (a single copy of the 16S rRNA, a single copy of the 23S rRNA, and two copies of the 5S rRNA), 1 tmRNA, and 1,911 CDS including 74 hypothetical genes, and 84 pseudogenes (Table S1). The minimum, maximum, and N50 sequence lengths of the CDS were 200 bp, 86,576 bp, and 26,755 bp, respectively. The total length and number of CDS are consistent with *L. delbrueckii* subsp. *indicus* JCM 15610^T^ which contained 2.022 million bases, and 1,956 CDS (6). The antibiotic resistance gene was not identified in the TY-11 genome.

### The prophages of the isolate TY-11

Using raw genome and metagenome assemblies as input, the stand-alone command-line program Phigaro can identify prophage areas (7). One Siphoviridae and three Myoviridae prophages were present in the TY-11 genome (Table S2). This data matched the number of prophages of *Lactobacillus delbrueckii* subsp. *indicus* JCM 15610^T^ (6).

### Phylogenetic study and identification of the isolate TY-11

#### Phylogenetic study

A phylogenetic study was conducted to draw a network of relationships within the species, *L. delbrueckii*. The new genome TY-11 was at the bottom of the tree, and the three most closely related unspecified subspecies genomes are mentioned as follows:

GCF_025186005.1 *Lactobacillus delbrueckii* = *Lactobacillus delbrueckii* strain 862, GCF_025193525.1 *Lactobacillus delbrueckii* = *Lactobacillus delbrueckii* strain CIRM-BIA 865, and GCF_021600505.1 *Lactobacillus delbrueckii* = *Lactobacillus delbrueckii* strain ME-792.

**Fig 3:**
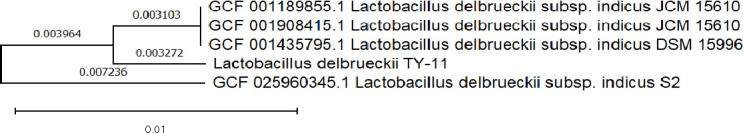
Phylogenetic tree-2. The tree was inferred using the neighbor-joining method employing five genomes of *L. delbrueckii* subsp. *indicus* (based on aligned amino-acid sequences of 353 common genes of the TY-11 genome and 4 subsp. *indicus* genomes).

The next nearest were three genomes of *L. delbrueckii* subsp. *indicus*. These seven genomes were organized in a cluster with a rather long branch to the rest of the tree. It was assumed that all seven are probably subsp. *indicus*. In the first tree, there were a total of four *Lactobacillus delbrueckii* subsp. *indicus*, except the TY-11 genome. The nearest three sequences of *Lactobacillus delbrueckii* subsp. *indicus* (GCF_001908415.1 = JCM 15610, GCF_001435795.1 = DSM 15996, GCF_001189855.1 = JCM 15610) stayed in a cluster. GCF_025960345.1 = S2 was placed outside of this cluster. This could be a misclassification (Fig. S1; Table S3). In the second tree, it showed the same feature, that is, GCF_025960345.1 = S2 was in the out-group and the other three subsp. *indicus* genomes stayed in one cluster near TY-11 (Fig. 3). Overall, from the tree of 262 genomes, it seems that the TY-11 belongs to *Lactobacillus delbrueckii* subsp. *indicus*.

#### Subspecies-specific identification by genotype and phenotype

1. Within species, Average Nucleotide Identity (ANI) values are larger than 97% in most cases (8). The draft genome of TY-11 showed 98.940% ANI with that of the closest microorganism, *Lactobacillus delbrueckii* subsp. *indicus* JCM 15610 (GCA_001189855.1, ASM118985v1). The strains of six subspecies, *delbrueckii, lactis, bulgaricus*, *indicus, sunkii*, and *jakobsenii* represented a little variation in the ANI percentage (Table S4).
2. The 16S sequence from the draft genome of TY-11 demonstrated 99.87% identity with that of the closest microorganism, *Lactobacillus delbrueckii* subsp. *indicus* JCM 15610.
3. The core genome sequence (353 genes) of TY-11 showed 99.01441% similarity with the cluster of all three nearest sequences of *L. delbrueckii* subsp. *indicus* (GCF_001908415.1 = JCM 15610, GCF_001435795.1 = DSM 15996, GCF_001189855.1 = JCM 15610), while it showed 98.32962% similarity with the second nearest *L. delbrueckii* subsp. *indicus* (GCF_025960345.1 = S2). Therefore, TY-11 was identified as *L. delbrueckii* subsp. *indicus*.
4. Subsp. *indicus*-specific genes were identified in the TY-11 genome. While the TY-11 genome lacked the proline iminopeptidase (pepIP) gene, which distinguished it from the positive *L. delbrueckii* subsp. *bulgaricus* and *lactis*, it contained the gene for recombinase RecA (LPJCKDFM_01499) and chaperonin GroEL (also known as Hsp60) (LPJCKDFM_00450) (Table S1), which are commonly present in subsp. *indicus* (4).
5. Its phenotypic characteristics set it apart from the non-lactose fermentative subsp. *delbrueckii*, *sunkii*, and *jakobsenii*. Lactose degradation was found constitutive in the TY-11 strain but it is regulated in subsp. *lactis*. Subsp. *bulgaricus* is also able constitutively to ferment lactose but unlike subsp. *bulgaricus,* the TY-11 strain was negative for b-galactosidase (details in Discussion Section). Analyzing functional genes and phenotypic characters of lactose fermentation, the TY-11 strain was identified as *L. delbrueckii* subsp. *indicus*.
6. According to genomic functional analysis, it was homofermentative, and its metabolic pathway was identified to produce L-lactic acid exclusively (details in Section Anaerobic Growth and Discussion Section).
7. According to the gram staining result, it was a gram-positive rod-shaped bacteria.

### Functionality analysis of the TY-11 genome by KEGG annotations

According to the KEGG Mapper Reconstruction Result, a total of 1,079 genes (56.5%) were annotated from 1,911 CDS. The genes (56.5%), annotated by KEGG annotations, were cited in this study as KEGG ID. The remaining genes (43.5%) from PROKKA (version 1.13) annotation were cited in this study mentioning PROKKA ID (9).

#### Central carbohydrate metabolism

The TY-11 carried a complete set of genes (11 genes) of the Embden-Meyerhof pathway for glycolysis to convert glucose to pyruvate (M00001). It had a complete set of genes (six genes) for the glycolysis core module involving three-carbon compounds (M00002). It gained the pentose phosphate pathway, oxidative phase, to convert glucose 6P to ribulose 5P (complete three genes, M00006) (Table S5). Phosphoribosyl 1-pyrophosphate (PRPP) biosynthesis (ribose 5P => 5 PRPP by ribose-phosphate pyrophosphokinase, K00948) was an additional feature of the TY-11 genome (M00005).

#### Anaerobic growth

The isolate TY-11 was an anaerobic bacterium according to genomic data because it had genes for the anaerobic metabolism. It carried the lactose dehydrogenase gene, L-lactate dehydrogenase (K00016), by which pyruvate is converted into L-lactate. One of the H transfer (oxidoreductase) enzymes, lactate dehydrogenase uses NADH to catalyze the reversible conversion of pyruvate to lactate. The enzyme essentially participates in the anaerobic metabolism of glucose when oxygen is absent or scarce (10).

Pyruvate + NADH + H+ --> Lactate + NAD+

The anaerobic ribonucleoside-triphosphate reductase activating protein (LPJCKDFM_00962) found in the TY-11 genome is activated under anaerobic conditions by the production of an organic free radical, which uses reduced flavodoxin and S-adenosylmethionine as cosubstrates to generate 5′-deoxy-adenosine (11). It also had anaerobic ribonucleoside-triphosphate reductase (LPJCKDFM_00963 = K21636), which has an oxygen-sensitive activity that reduces CTP to dCTP in the presence of NADPH, dithiothreitol, Mg2+ ions, and ATP (12). The anaerobic character of the isolate TY-11 is consistent with the anaerobic environment in the human gut because 99.9% of colonic microflora are obligate anaerobes (13).

#### Lactose metabolism and lactose intolerance

According to the present study, the TY-11 had a lactose-transporting PTS system (K02786 and K02788) (discussed in Section Transport System), which simultaneously transports lactose from the periplasm or extracellular space into the cytoplasm and phosphorylates it. The TY-11 also had 6-phospho-beta-galactosidase (lacG = K01220), which produces glucose and galactose-6-phosphate (Gal-6P) during the hydrolysis of lactose-6-phosphate (Lac-6P) (14, 15). This PTS system (K02786 and K02788) and lacG appear to be rare in the *lactobacilli* of the acidophilus group and lactobacilli in general.

In the TY-11 genome, lactose permease enzyme for lactose transportation failed to appear, although it retained probable lactose utilizing protein: glycoside hydrolase family 1 protein (LPJCKDFM_01304 = K01223), glycoside hydrolase family 68 protein, glycoside hydrolase (LPJCKDFM_00424), and glycoside hydrolase family 73 protein (LPJCKDFM_00227), which are the enzymes with a number of known activities including beta (EC 3.2.1.23) galactosidase (16).

#### Adaptation to heat shock stress and cold shock stress

The TY-11 beard various genes encoding heat stress proteins, for example, Hsp33 family molecular chaperone HslO (LPJCKDFM_00136), molecular chaperone DnaJ (LPJCKDFM_01166), molecular chaperone DnaK (K04043), chaperonin GroEL (K04077), and co-chaperone GroES (LPJCKDFM_00449) (17). GroEL and GroES are proteins, which are required for the proper folding of many proteins at different temperature conditions. In *Lactobacillus delbrueckii* subsp. *bulgaricus*, it was shown that DnaK has a role at high temperatures (18). This genome had a gene (LPJCKDFM_01163) for the heat shock repressor protein HrcA. It had a defense mechanism against sudden heat shock stress (19). It had a cold shock domain-containing protein (LPJCKDFM_00078). These so-called “cold shock” proteins are thought to help the cell to survive in temperatures lower than the optimum growth temperature, by contrast with heat shock proteins, which help the cell to survive in temperatures greater than the optimum (20).

#### Tolerance to weak acids and bile salts in the gut

The TY-11 genome had bile salt hydrolase (BSH)/choloylglycine hydrolase (K01442). These proteins are associated with tolerance to acids and bile salts in the gut. Certain species of the indigenous microflora, including a number of *lactobacilli* and *bifidobacteria*, have evolved the ability to deconjugate bile salts by bile salt hydrolase (BSH) (21).

#### pH homeostasis under acid stress

The TY-11 had F0F1 ATP synthase subunits A, B, C, alpha, beta, gamma, delta, and epsilon (LPJCKDFM_00033–40). Low driving force causes ATP synthases to act as ATPases and produce a transmembrane ion gradient at the expense of ATP hydrolysis (22). This genome had chaperonin GroEL (K04077) and co-chaperone GroES (LPJCKDFM_00449). These general stress proteins are overexpressed during acid stress (23).

#### Lifestyle adaptation to other stress

The TY-11 genome coded for glutaredoxin (LPJCKDFM_00958) and thioredoxin-disulfide reductase (LPJCKDFM_01338 = K00384), which catalyze glutathione-dependent disulfide reductions. These genes may help the organism withstand oxidative stress. The TY-11 genome contained genes that code for stress-related proteases like the ATP-dependent Clp protease ATP-binding subunit ClpX (LPJCKDFM_00107) and proteolytic subunit clpP (LPJCKDFM_00518 and LPJCKDFM_01329) and subunit HslV (LPJCKDFM_00980), which prevent abnormal protein damage (17). It had genes for osmoprotectant transport system permease protein (LPJCKDFM_00370, LPJCKDFM_00931, and LPJCKDFM_00933 = K05846) which play crucial roles in the adaptation of cells to various adverse environmental conditions (24). Peroxide stress protein YaaA (LPJCKDFM_00708) was predicted to be involved in the cellular response to hydrogen peroxide (H_2_O_2_) stress. By reducing the amount of unincorporated iron in the cell, YaaA protects DNA and proteins from oxidative damage caused by H_2_O_2_ stress (11).

#### Biosynthesis system of amino acid

A complete threonine pathway, employing five genes (M00018), was predicted in the TY11 genome: aspartate → homoserine →threonine. It had the gene sets to complete the pathway for proline metabolism (three genes for M00015) (Table 2).

**TABLE 2:** Threonine and proline metabolism

Name of pathway	Prokka ID	KEGG orthologs	Gene name	Gene symbol
(M00018) The synthesis of threonine, aspartate → homoserine → threonine	LPJCKDFM_01420	K00003	Homoserine dehydrogenase [EC:1.1.1.3]	hom
	LPJCKDFM_01422	K00133	Aspartate-semialdehyde dehydrogenase [EC:1.2.1.11]	asd
	LPJCKDFM_00621	K00872	Homoserine kinase [EC:2.7.1.39]	thrB
	LPJCKDFM_00284, LPJCKDFM_01421	K00928	Aspartate kinase [EC:2.7.2.4]	lysC
	LPJCKDFM_00622	K01733	Threonine synthase [EC:4.2.3.1]	thrC
(M00015) Proline biosynthesis, glutamate => proline	LPJCKDFM_00742	K00147	Glutamate-5-semialdehyde dehydrogenase [EC:1.2.1.41]	proA
	LPJCKDFM_00741	K00286	Pyrroline-5-carboxylate reductase [EC:1.5.1.2]	proC
	LPJCKDFM_00743	K00931	Glutamate 5-kinase	proB

**Fig 4:**
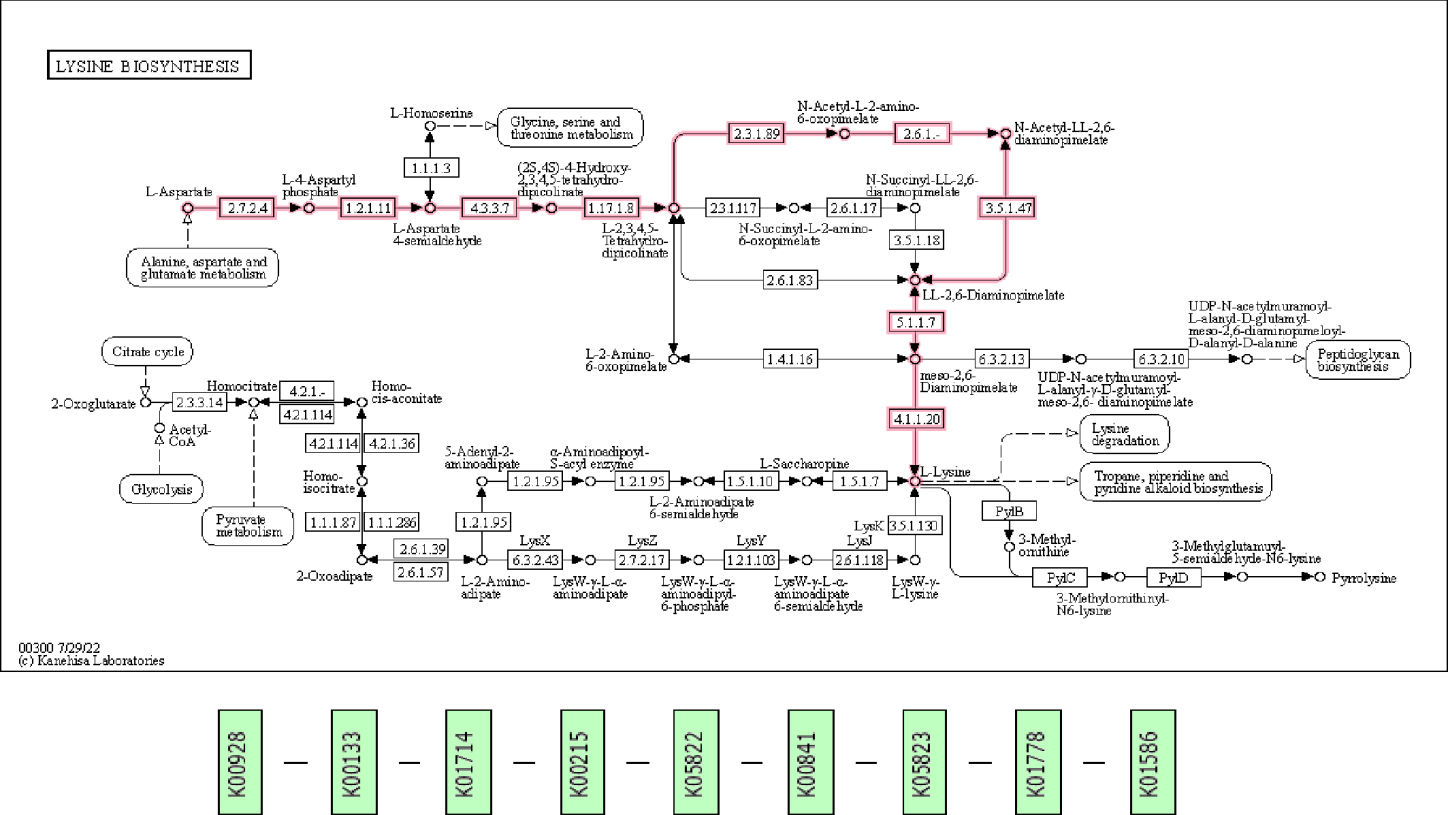
Lysine biosynthesis. The upper flowchart shows the lysine biosynthesis pathway of the TY-11 genome in purple color. The KEGG orthologs of the enzymes involved in the lysine biosynthesis pathway (upper flowchart) are represented serially from the left to the right in the lower flowchart. Reproduced with permission from Kanehisa Laboratories.

**Fig 5:**
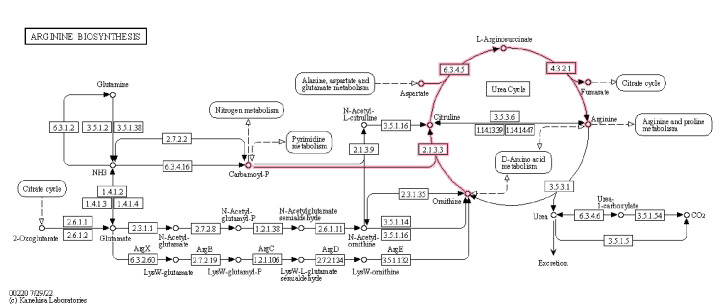
Arginine biosynthesis. The diagram depicts the arginine biosynthesis pathway of the TY-11 genome in purple color. The enzymes involved in arginine biosynthesis are as follows: 21.33 = K00611, 63.45 = K01940, and 43.21 = K01755. Reproduced with permission from Kanehisa Laboratories.

This probiotic had 12 genes to complete the entire acetyl-DAP pathway for lysine metabolism from aspartate (K000300, M00525): K00928, K00133, K01714, K00215, K05822, K00841, K05823, K01778, K01586, and K05825 (Fig. 4). Lysine is an essential amino acid for humans (25). It had the complete gene sets for arginine metabolism (K000220, M00844): K00611, K01940, and K01755 (Fig. 5). Arginine is a conditionally essential amino acid for humans (26). It possessed a better transport system to make up for its restricted ability to synthesize amino acids (17).

#### Metabolism of cofactors and vitamins

The TY-11 genome had four genes encoding enzymes for Coenzyme A biosynthesis (M00120) from pantothenate. All genes for folate biosynthesis pathway were annotated in the TY-11 genome (Ko:00790) to synthesize folate from Guanosine-5′-triphosphate (GTP) except alkaline phosphatase (EC: 3.1.3.1). The TY-11 genome entailed dihydrofolate reductase (K00287), which converts folate to dihydrofolate, and then dihydrofolate to tetrahydrofolate (THF) (Table S6). In cells, folic acid (vitamin B9) is reduced to THF, a biologically active form (27). The folate deficiency manifests in fatigue, muscle weakness, tingling of extremities, and loss of joint position/coordination in humans (28, 29).

#### Transport system

The ABC transporter system and PTS were represented by 78 genes in the TY-11 genome, which was around 1.9 Mb in size. The genome contained 62 genes that coded for the elements of the ABC transporter system (Table S7). ABC transporter proteins are by far the most abundant of transporters, typically accounting for half of the transporters in a bacterial genome (30).

Many of the ABC importers of this TY-11 genome transport inorganic ions, amino acids, and peptides; however, it is unknown what kinds of substances the majority of the exporters export. The genome contained 11 amino acid-permease type transporters and one transporter for branched-chain amino acids, which is the typical number of transporters found in the majority of other lactic acid bacteria (6, 17). The genes encoding the flippase proteins (LPJCKDFM_01660 flippase-like domain-containing protein, LPJCKDFM_01863&00849 flippase, and LPJCKDFM_00212 oligosaccharide flippase family protein) were also discovered in the TY-11 genome. Flippases, which are infrequently written flipases, are ABC transporter or P4-type ATPase families of transmembrane lipid transporter proteins that are found in membranes. They help phospholipid molecules flow across the two leaflets that make up a cell’s membrane (31).

The strain TY-11 had an osmoprotectant transport system permease protein (K05846). Osmoprotectants (also termed compatible solutes) are highly soluble compounds that carry no net charge at physiological pH and are nontoxic at high concentrations. Osmoprotectants play a vital role in helping cells adapt to a variety of harmful environmental situations (17, 30).

The PTS is a major carbohydrate active transport system in bacteria that catalyzes the phosphorylation of sugar substrates to cross the microbial cell membrane. In all, 16 genes of the TY-11 were related to the PTS. It had Phosphoenolpyruvate-protein phosphotransferase (ptsI encoding PTS system enzyme I) (EC:2.7.3.9). In the first step of the PTS system, this enzyme phosphorylates the histidine-containing phosphocarrier protein (HPr). Phosphoryl groups from phosphoenol-pyruvate are delivered by HPr to EII enzymes (EIIs), which are also components of the TY-11 PTS (32). Beta-glucosides, cellobiose, fructose, glucitol, galactose, lactose, mannose, sorbitol, and glucose were among the 13 phosphoenolpyruvate-dependent PTS EII complexes connected to the transport of carbon sources that were encoded by this genome. The capacity of the TY-11 for transporting carbon is increased by these sugar PTSs’ ability to import several substrates.

#### Secretion system

The TY-11 genome featured the secretion system Sec-SRP. This system includes the signal recognition particle subunit SRP54 (K03106), fused signal recognition particle receptor (K03110) as well as the preprotein translocase subunit SecA (K03070), SecY (K03076), SecG (K03075), and SecE (K03073). Apart from that, the genome contained membrane protein YidC homologs (LPJCKDFM_00904, LPJCKDFM_01385 = K03217), which function for the insertion of hydrophobic regions into the lipid bilayer (33). The secretion system of this genome also consisted of the preprotein translocase component YajC (K03210). The TY-11 genome lacked the genes encoding the Tat-dependent protein transport pathway.

#### Extracellular matrix, immunity, and adhesion

Exopolysaccharides (EPS) are extracellular matrix excreted as tightly attached capsules or loosely bound outer layer slime in microorganisms. It has been proven that exopolysaccharides have a role in the way bacteria communicate with their surroundings. They have been demonstrated to be involved in bacterial biofilm development, adherence to abiotic elements and biotic substrates, and immune system activation (17). In the TY-11 genome, the EPS biosynthetic gene clustered from UDP-galactopyranose mutase (K01854), seven enzymes of glycosyltransferases (ko:01003), and one exopolysaccharide biosynthesis protein (LPJCKDFM_00840).

Another point to consider is the TY-11 retained a gene for UDP-GlcNAc:undecaprenyl-phosphate/decaprenyl-phosphate GlcNAc-1-phosphate transferase (K02851). N-acetyl-alpha-D-glucosaminyl-diphospho-ditrans, octacis-undecaprenol is a key lipid intermediary for the biosynthesis of numerous microbial cell envelope components (cell wall, cytoplasmic membrane, and capsule of gram-positive bacteria), and K02851 catalyzes its synthesis.

In some gram-positive bacteria, the enzyme also starts the manufacture of teichoic acid (34). The wall teichoic acids (WTAs) are teichoic acids that are attached to the lipid membrane (35). For teichoic acid synthesis (00552 Teichoic acid biosynthesis), the TY-11 genome contained 12 genes. Lipoteichoic acids (LTAs) are teichoic acids that are covalently attached to peptidoglycan (35). Biosynthesis of d-alanyl-lipoteichoic acid requires enzymatic activation of D-alanine for its incorporation into the membrane-associated polymer (mLTA). The TY-11 genome had all five genes of the dlt operon for the biosynthesis of d-alanyl-lipoteichoic acid: teichoic acid D-Ala incorporation-associated protein DltX (LPJCKDFM_01587), D-alanine-poly (phosphoribitol) ligase subunit DltA (K03367), protein DltB (K03739) involved in the production of D-alanyl-lipoteichoic acid, D-alanine--poly(phosphoribitol) ligase subunit DltC (K14188), and D-alanyl-lipoteichoic acid biosynthesis protein DltD (K03740) (Table S9). Lipoteichoic Acid (LTA) stimulates nuclear transcription factor kappa B, a component of the innate immune response, after binding to membrane-bound Toll-like receptor 2, according to Nguyen et al. (36).

#### Flavour-related activities

Chemicals that influence the sensations of taste and odor play a major role in determining flavor, which is the sensory perception of food or other things. Glycolysis, proteolysis, and lipolysis are a few of the biochemical procedures used by LAB to create flavor compounds. The flavor-related activities of LAB mostly depend on the species (37). The flavor is an important feature of a probiotic to use for functional food. The TY-11 genome possessed pathways of these three types’ metabolisms for producing flavor. For this reason, the TY-11 can be used for preparing probiotic yogurt or any other fermented food.

1. Glycolysis: Basically, the only form of anaerobic homofermentation that the TY-11 genome was able to perform was homolactic fermentation (see Section Lactose Metabolism and Lactose Intolerance and Discussion Section for details), which facilitates the taste of lactic acid, but not acetate and acetaldehyde.
2. Proteolysis: The TY-11 genome carried the genes for proteolysis, an important biochemical pathway of flavor production (discussed in Section Characterization of Antimicrobial Activity from Genomic Study—iv). Moreover, transporter proteins for proteolysis, encoded by this genome, were identified as oligopeptide transporters, amino acid branched-chain transport system substrate-binding proteins, ion-linked transporters for peptides, and ATP-driven transporters for peptides (discussed in Section Transport System, Table S7). Bitter peptides are generally produced during proteolysis (37).
3. Lipolysis: The TY-11 genome harbored genes for thioesterase (LPJCKDFM_00442), metallophosphoesterase (LPJCKDFM_00966), DHH family phosphoesterase (LPJCKDFM_01373), glycerophosphodiester phosphodiesterase family protein (LPJCKDFM_01556 = K01126), and phospholipase D-like domain-containing protein LPJCKDFM_01880) for producing flavor compounds from lipids. An ester bond is hydrolyzed by esterases to produce an alcohol and a carboxylic acid. Carboxylic acids, like other acids, often taste sour. There are many distinct flavors of alcohol (37, 38).

#### Recovering digestive problems within the human intestine

The strain TY-11’s encoded genes were thought to be appropriate for the human gut to break down proteins, fats, carbs, and other nutrients:

1. Carbohydrate metabolism: Its genes for lactose fermentation might be utilized to reduce lactose intolerance (details in Section Lactose Metabolism and Lactose Intolerance and Discussion Section). Lactose intolerance is a major problem globally.
2. Protein catabolism: Dietary peptides, free amino acids (FAAs), and protein that are ingested enter the large intestine where they are further fermented by the diverse gut flora. The strain TY-11 encoded genes for proteolytic enzymes (discussed in Section Characterization of Antimicrobial Activity from Genomic Study—iv) and peptide transporters (discussed in Section Transport System) to absorb and catalyze small amino acids and peptides. It might affect the host’s energy balance through the anaerobic metabolism of peptides and proteins.
3. Metabolism of toxic metabolites: Some harmful metabolites can be produced from proteins in the human intestine. This probiotic possessed genes for multicopper oxidase domain-containing protein (LPJCKDFM_00777) and nitroreductase (LPJCKDFM_00111) enzymes to decrease the toxic metabolites (discussed in Section Identifying Genes for the Most Prevalent Harmful by-Products of LABs).
4. Lipid catabolism: This strain was a source of esterases (Section Flavor-Related Activities) and the transmembrane lipid transporter protein (flippases) (Section Transport System) to contribute to modulating human adiposity (39).

#### Identifying genes for the most prevalent harmful by-products of LABs

It is important to screen for the typical bacterial toxic metabolites that are dangerous to human health and present in LABs. These involve important enzymes, such as D-lactic acid (D-lactate), hemolysins, and biogenic amines (BAs) produced by amino acid decarboxylase (40). Although BAs have beneficial impacts on how the body functions, an excessive amount of BAs can be harmful and cause diarrhea, food poisoning, vomiting, sweating, or tachycardia. In addition, they can hasten the development of cancer. Histamine (HIS), tyramine (TYR), putrescine (PUT), cadaverine (CAD), tryptamine (TRP), -phenethylamine (PHE), spermidine (SPD), and spermine (SPM) are the most prevalent BAs noticed in cultured foods and drinks. Decarboxylase or deiminase from microorganisms operates to create BAS (41). The TY-11 genome did not contain any gene for producing these metabolites. This is consistent with the biochemical character of *Lactobacillus delbrueckii* subsp. *indicus* WDS-7 (5).

More importantly, the TY-11 genome carried a multicopper oxidase domain-containing protein (LPJCKDFM_00777). Multicopper oxidase (MCO)-mediated BAS degradation is an auspicious method as the fermentation process, food nutrition, and flavor are unaffected (42). This genome also contained nitroreductase (LPJCKDFM_00111), a flavoenzyme that catalyzes the reduction of the nitro groups on nitroaromatic and nitroheterocyclic compounds in a NAD (*P*)H-dependent manner. For living things, the most nitroaromatic chemicals are harmful and mutagenic; however, certain microbes like the strain TY-11, evolved oxidative or reductive mechanisms to break down or modify these substances. Due to its promising uses in bioremediation, biocatalysis, and biomedicine, particularly in prodrug activation for chemotherapeutic cancer therapies, nitroreductases have attracted a lot of attention (40, 43).

#### Characterization of antimicrobial activity from genomic study

The generation of inhibitory chemicals (such as bacteriocin, hydrogen peroxide, and acid), blocking of adhesion sites, and competing for resources are only a few of the ways that probiotics inhibit both gram-negative and gram-positive pathogenic bacteria (44). The TY-11 genome had genes for the following antagonistic mechanism:

1. Three genes commonly are considered to contribute to H_2_O_2_ production, for example, pyruvate oxidase, NADH oxidase, and lactate oxidase (45). Pyruvate oxidase (K00158) was predicted in the TY-11, although NADH oxidase and lactate oxidase were not identified. In enzymology, pyruvate oxidase (EC 1.2.3.3) catalyzes the reaction as follows:

$Pyruvate+Phosphate+O_{2}⇌Acetyl\;Phosphate+CO_{2}+H_{2}O_{2}$

This enzyme has pyruvate, phosphate, and oxygen as its three substrates; acetyl phosphate, carbon dioxide, and hydrogen peroxide are its three by-products. The aforementioned enzyme is a member of the oxidoreductase family, especially those that operate on aldehydes or oxo-donor molecules with oxygen as the acceptor (46).

- The gene clusters involved in EPS production, which are present in the TY-11 genome, aid in the attachment of probiotic cells to gut epithelial cells, and by blocking adhesion sites, prevent pathogens from adhering to the gut. EPS biosynthesis is discussed in Sections Extracellular Matrix, Immunity, and Adhesion and Flavor-Related Activities.
- Because of its superior nutritional (carbohydrate, amino acid, vitamin, mineral, etc.) metabolism and transport ability, the strain TY-11 was assumed to be able to compete with pathogens. These capabilities were discussed in Sections Central Carbohydrate Metabolism, Biosynthesis System of Amino Acid, Metabolism of Cofactors and Vitamins, and Transport System.
- Two benefits of the acid produced by LABs include lowering the pH of the gastrointestinal tract and inhibiting the growth of harmful bacteria. The enzyme genes found in the isolate TY-11 that regulate post-acidification are (1) genes related to lactose metabolism and the pyruvate biosynthesis pathway (explained in Section Central Carbohydrate Metabolism); (2) the F1F0-ATPase gene cluster (discussed in Section Transport System); (3) genes related to basic amino acid metabolism and synthesis (details in Sections Biosynthesis System of Amino Acid and Discussion Section); (4) genes related to proteins involved in ion transport (discussed in Section Transport System); (5) genes related to enzymes involved in the biosynthetic metabolic pathway of cell membranes (discussed in Section Extracellular Matrix, Immunity, and Adhesion); and key protease genes in the proteolytic system; among others. A proteolytic enzyme, also known as a protease, proteinase, or peptidase, is any one of the categories of enzymes that break down proteins into smaller pieces known as peptides and then into their amino acid constituents. The TY-11 contained genes for proteolytic enzymes for generating peptides and releasing free amino acids to satisfy its nutritional needs (47). The primary proteolysis system, that provided the TY-11 its capacity for producing acidity, was controlled by a total of 30 genes (Table S10).

### Mining bacterial genomic DNA for bacteriocins

**Fig 6:**
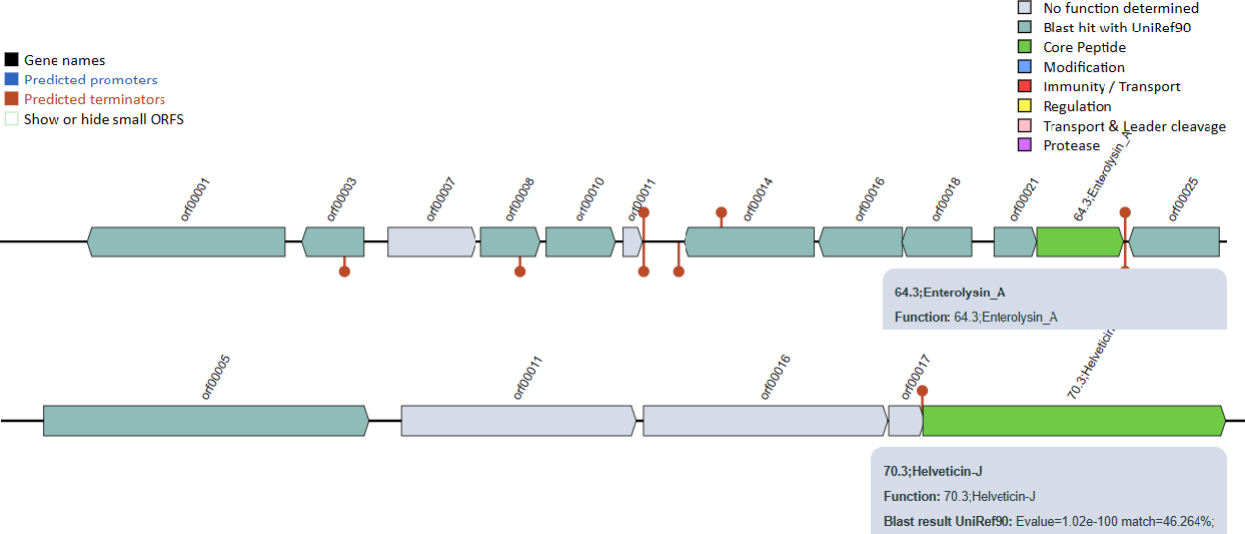
Two bacteriocins in the TY-11 genome: Helveticin-J (331 bp) and Enterolysin A (275 bp).

To prevent the proliferation of bacterial strains that are identical to or extremely close to them, bacteria create toxins called bacteriocins (48). Core peptides and their associated proteins were found in the TY-11 genome for synthesizing two bacteriocins. Helveticin-J (331 bp with 46.26% match) of the TY-11 showed 99.09% identity and 93% query coverage with helveticin J family class III bacteriocin (*Lactobacillus delbrueckii*) of blast search. Enterolysin_A (275 bp with 60.49% match) was also predicted in the TY-11 genome (Fig. 6). A total of 94 *Lactobacillus* genomes carry Helveticin-J, and 48 *Lactobacillus* genomes express Enterolysin_A, based on UniProtKB search results (11).

### Genomic comparisons

#### The TY-11 Entire Genome Represented by *Lactobacillus delbrueckii* Core Genome, TY-11 Non-Core Genome, and subsp. *indicus* Core Genome

**Fig 7:**
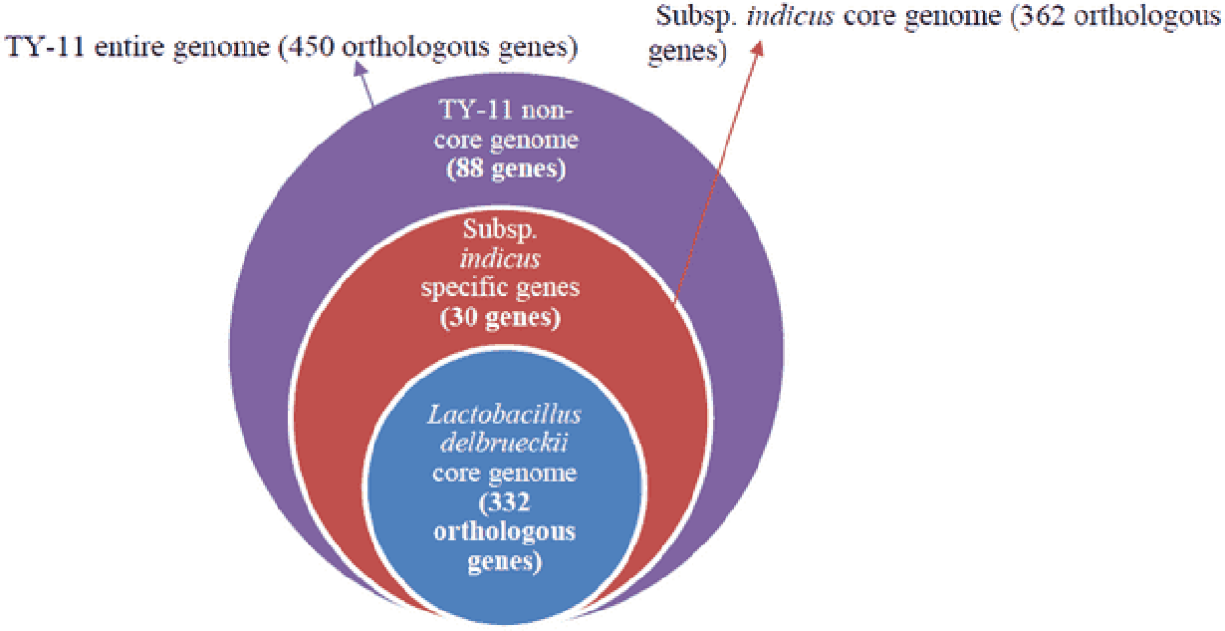
The Venn diagram for the draft entire genome of the strain TY-11 (450 orthologous genes). The TY-11 entire genome (450 orthologous genes) had been represented by *Lactobacillus delbrueckii* core genome (332 orthologous genes) and subsp. *indicus* core genome (362 orthologous genes).

The TY-11 entire genome had a total of 450 annotated orthologue genes (Table S11). In all, 332 orthologous genes were defined as *Lactobacillus delbrueckii* core genome (263 genomes), and 362 orthologous genes were calculated as subsp. *indicus* core genome (five genomes). In total, 30 genes (362 genes of subsp. *indicus* core genome − 332 genes of *Lactobacillus delbrueckii* core genome = 30 genes) were predicted as core genes of subsp. *indicus* (five genomes) but not core *Lactobacillus delbruickii* genes. These 30 genes (computed within 263 genomes at a 95% threshold) are subsp. *indicus*-specific genes of the TY-11 genome. The remaining 88 genes (450 annotated orthologous genes of our isolated entire genome − 362 genes of subsp. *indicus* core genome = 88 genes) were the TY-11 non-core genome (Table S12; Fig. S1; Fig. 7).

The TY-11 non-core genome was further annotated with the KEGG database, where 64 genes out of 88 genes were annotated (72.7%). Protein families: genetic data processing (eight genes), metabolism of carbohydrates (11 genes), and protein families: signal and cellular functions (12 genes) accounted for the majority of the genes (Table S12). The TY-11 non-core genome was composed of 13 genes (out of 88 non-core genes) encoding for CRISPR/CRISPR-associated system (Cas) genes, which were not annotated with the KEGG database (Table S12). Of the 13 CRISPR/Cas genes in the TY-11 genome, 11 genes (red marked in Table S12) were completely lacking in the other 4 genomes of the subspecies *indicus*. Another six genes of the TY-11 genome that were completely missed in the other four genomes of the subspecies *indicus* were as follows: alkaline shock response membrane anchor protein AmaP (LPJCKDFM_01933), mid-cell-anchored protein Z (LPJCKDFM_00954), GTP cyclohydrolase II (LPJCKDFM_01936), alpha,alpha-phosphotrehalase (LPJCKDFM_00870), trehalose operon repressor (LPJCKDFM_00871), and putative protein YqbN (LPJCKDFM_00321). In total, the TY-11 genome carried 17 unique genes out of 88 non-core genes.

#### Comparisons between subsp. *indicus* and *bulguricus* core genomes

**Fig 8:**
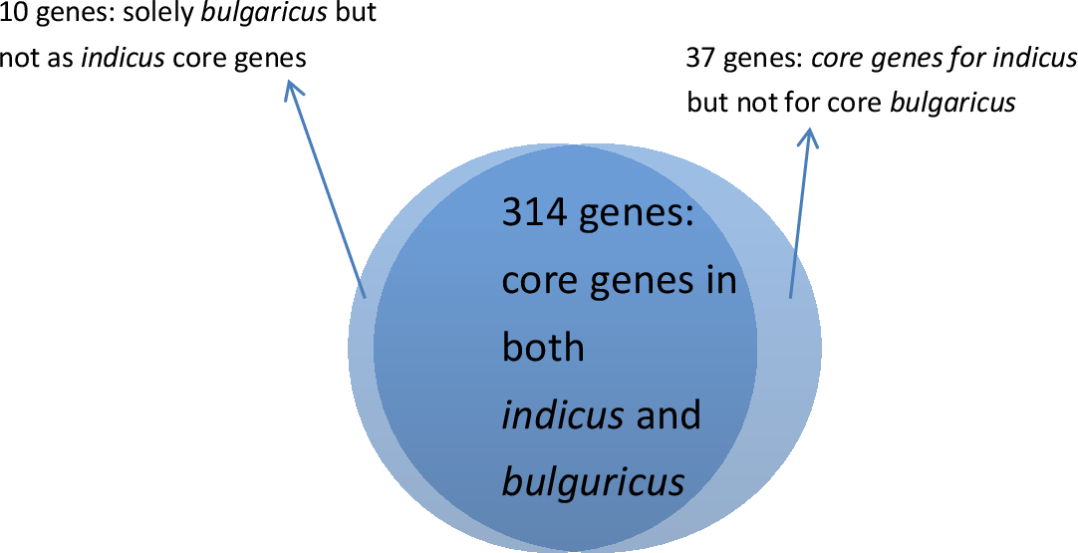
A Venn diagram for comparisons between subspecies *indicus* and *bulguricus* core genomes.

One of the most well-known probiotics, *Lactobacillus delbrueckii* subsp. *bulgaricus* (formerly known as *Lactobacillus bulgaricus*), is the principal bacteria utilized in the manufacturing of cheeses, yogurts, as well as other procedures including naturally fermented goods (6, 18, 38). In this study, we computed the core genome of subsp. *bulgaricus* with 69 genomes*,* and *indicus* with five genomes (Fig. S1; Table S3). A 95% threshold was used to compute the core genome. We computed 324 and 351 core genes for the subspecies *bulgaricus* and *indicus*, respectively. Among these genes, only 314 genes were common between the subspecies *bulgaricus* and *indicus* (Fig. 8; Table S13).

Only 10 core genes were annotated for “solely *bulgaricus* but not as *indicus* core genes” (Table S14). On the contrary, 37 core genes were predicted only for “at least 95% *indicus* but not for core *bulgaricus”* (Table S13). The TY-11 genome entailed all 37 genes for its function.

### The phenotype tests of the isolate TY-11

#### Antimicrobial activity test

**Fig 9:**
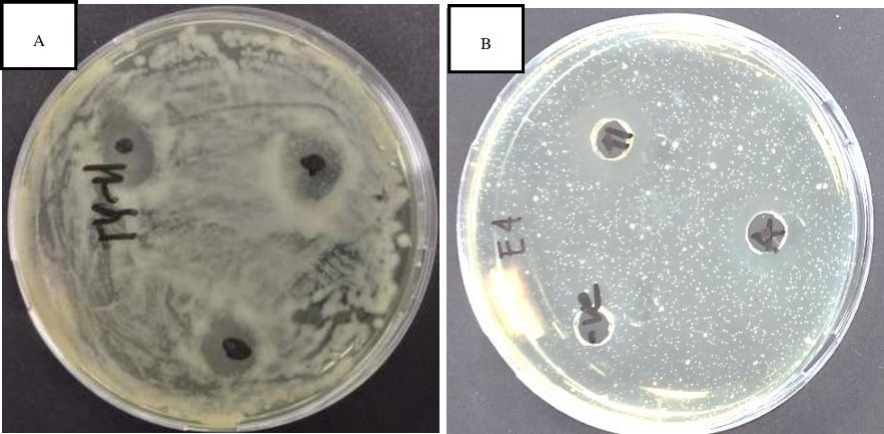
The photograph shows that *Escherichia coli* ATCC 8739 was inhibited by the isolated strain TY-11. In picture A, all three inhibition zones indicate the antimicrobial activity of the isolate TY-11 against *E. coli* by a spot test. In Image B, Inhibition Zone-11 shows a larger inhibition zone than Inhibition Zone-A. The antibacterial activity of isolate TY-11 is shown in Inhibition Zone-11 against *E. coli* by an agar well diffusion method. *Lactobacillus acidophilus* (LA1063), the control, has antimicrobial action in Inhibition Zone-A.

In our study, the strain TY-11 demonstrated an inhibition against *Escherichia coli* ATCC 8739 according to the spot test. Measuring the ZOI was not possible due to the irregular clear zone which was caused by the uncontrolled flow of the culture broth supernatant on the agar plate (Fig. 9). Additional testing, like the agar well diffusion method, would be ideal for determining the ZOI. We utilized this method in this study against *Escherichia coli* ATCC 8739.

When the pH of the TY-11 culture broth supernatant was 3.97 ± 0.01, we observed that the inhibition zone diameter against *E. coli* was 20.25 ± 0.35 mm. This result indicates that the strain TY-11 had a strong inhibition against the tested pathogen. At pH 4.17 ± 2.41 in the control culture broth supernatant, we measured the inhibitory zone diameter against *E. coli* which was 16.75 ± 0.35 mm. The isolate TY-11 had a slightly larger inhibitory zone diameter than the control.

#### Antibiotic sensitivity test

The basis for the antibiotic sensitivity test for *L. delbrueckii* strains must be *Lactobacillus* obligate homofermentative strains in accordance with CLSI guidelines. This includes the following dosages: ampicillin (10 µg/100 µL), vancomycin (30 µg/100 µL), gentamicin (10 µg/100 µL), kanamycin (30 µg/100 µL), streptomycin (10 µg/100 µL), erythromycin (15 µg/100 µL), tetracycline (30 µg/100 µL), chloramphenicol (30 µg/100 µL), tylosin (30 µg/100 µL), and clindamycin (10 µg/100 µL) (5, 49). As per the Clinical and Laboratory Standards Institute’s (CLSI) description of microbiological breakpoints for antibiotics: the isolates were classified as susceptible if they were 20 mm or more than 20 mm in diameter, intermediate if they were between 15 and 19 mm, and resistant if their zone of inhibition was less than or equal to 14 mm. The isolated strain TY-11 was susceptible to all the tested antibiotics at the above-mentioned concentrations (Table 3) (49). As a control *Lactobacillus acidophilus* (LA1063) was sensitive having a zone of inhibition greater than 20 mm against all antibiotics.

**TABLE 3:** Antibiotic sensitivity test

Antibiotic name	Antibiotic concentration	Diameter of ZOI (mm)
Ampicillin	10 µg/100 µL	49.67 ± 4.04
Vancomycin	30 µg/100 µL	39.17 ± 1.04
Gentamicin	10 µg/100 µL	28.0 ± 2.65
Kanamycin	30 µg/100 µL	20.0 ± 1.0
Streptomycin	10 µg/100 µL	23.0 ± 2.0
Erythromycin	15 µg/100 µL	34.5 ± 2.18
Tetracycline	30 µg/100 µL	44.0 ± 1.0
Chloramphenicol	30 µg/100 µL	41.33 ± 1.53
Tylosin	30 µg/100 µL	48.33 ± 4.04
Clindamycin	10 µg/100 µL	49.67 ± 0.58

## DISCUSSION

### Isolation and identification of the strain TY-11

In this study, the strain TY-11 had been isolated from native yogurt in Bangladesh, and identified as *Lactobacillus delbrueckii* subsp. *indicus* by its morphology, microscopic observation, 16S sequencing, biochemical test, ANI value of draft genome, and phylogenetic tree.

As far as we know, previously only one phylogenetic tree (maximum-likelihood tree) was constructed by the 689 core genes (amino acid sequences) from 31 genomes representing all six subspecies of *Lactobacillus delbrueckii* (6). Using the neighbor-joining approach, we created an extensive phylogenetic tree of 262 genomes, comprising a core of 332 genes. The genome TY-11 was positioned at the bottom of the tree (Fig. S1; Table S3), which was consistent with the findings of Baek et al. (6). In our study, the phylogenetic tree-1 differed from the previous work in that the genomes of various subspecies were placed scattered within the tree. This suggests that some strains belonging to different subspecies were probably misclassified. In the tree-1, when we studied the subs. *indicus*, every other subspecies was already an outgroup. In this regard, the tree truly included a large number of outgroup taxa, which was necessary to determine subsp. *indicus*’s position within the species. In this case, the evolution of the entire species was not necessarily the main focus of our investigation. Therefore, we did not include outgroups in the tree.

### Functional genomic study

According to our knowledge, only Baek et al. (6) conducted a comparative genomic study of a reference strain (JCM 15610^T^) of *Lactobacillus delbrueckii* subsp. *indicus* with other subspecies strains of *Lactobacillus delbrueckii*. However, the probiotic potential of subsp. *indicus*, which was covered for our isolate TY-11, was not included in the previous study (6). The TY-11 genome (1,916,674 bp) had the genetic features of an ideal probiotic to adapt to the variety of challenging settings found in the human gut. No genes were found for antibiotic resistance or hazardous compounds in this strain. It contained genes for adaptation to environmental stress conditions, metabolism of nutrients and toxic metabolites, recovering digestive disorders, secretion system, extracellular matrix, immunity, adhesion, flavor-producing capabilities, antimicrobial activities, and bacteriocin biosynthesis.

The isolate TY-11 harbored genes for glycolysis (Embden-Meyerhof pathway) (M00001), pentose phosphate pathway (M00006), and the three-carbon compound core module of glycolysis (M00002). When glucose is broken down into pyruvate, a small quantity of ATP (energy) and NADH (reducing power) are produced. This process is known as glycolysis. It is a key process that results in the crucial precursor metabolites glucose-6P and fructose-6P, which have six carbons, and glycerone-P, glyceraldehyde-3P, glycerate-3P, phosphoenolpyruvate, and pyruvate, which have three carbons (M00001). The chemical pathways of three-carbon molecules from glycerone-P to pyruvate comprise a conserved core module (M00002) in almost all organisms with fully sequenced genomes (50). The pyruvate dehydrogenase and other enzymes required for the transformation of pyruvate into acetaldehyde, acetyl-coenzyme, and acetate were absent in the strain TY-11. Since the human gut has an anaerobic environment, the absence of pyruvate dehydrogenase complex in the strain TY-11 makes sense (17). It was a homofermentative microorganism that could only produce homolactic fermentation. It is possible to separate Lactobacilli into two groups: (1) homofermentative species that produce mainly lactic acid (>65%) from glucose fermentation (e.g., *Lactobacillus delbrueckii* subsp. *indicus, L. acidophilus,* and *L. casei*) and (2) species of heterofermentation that generate lactic acid as well as significant amounts of acetate, ethanol, and CO2 (e.g., *L. fermentum*). Through the Embden-Meyerhof-Parnas route, homofermentative LAB preferentially uses hexoses (often glucose). The pentose phosphate pathway may be also present in some homofermentative lactic acid bacteria (LAB) (51). It has happened for the TY-11 genome.

Since homofermentative bacteria are typically employed as starting cultures in the dairy sector, the strain TY-11 is a potential probiotic to utilize for this purpose. By contrast, heterofermentative bacteria are rarely used as starter cultures within the dairy sector because they cause defects such as slits in hard cheeses and bloated packaging in other dairy products due to CO_2_ gas production (52).

The strain TY-11 lacked β-galactosidase (EC 3.2.1.23); however, like other strains of subsp. *bulgaricus*, it was able to constitutively digest lactose, which was proved by a biochemical test in our study (3). This evidence suggests that the strain possessed unique genetic equipment for lactose degradation, which differed from that of the other recognized subspecies. Characterization of this equipment was beyond the scope of the previous studies (4). lacG gene was found in 4 genomes including the strain TY-11 out of 5 genomes in subsp. *indicus,* while it was observed in 7 genomes out of 69 genomes in subsp. *bulguricus* that we used in the phylogenetic tree-1 (Fig. S1). This system is found active in *Lactobacillus delbrueckii* subsp. *lactis*. Glycoside hydrolase family genes found in this genome (functionally similar to β-galactosidase) have most probably been acquired in the ancestors of *L. delbrueckii* by horizontal gene transfer (53). Worldwide, lactose intolerance in humans is a big problem. Treatment of lactose intolerance in humans can include colonic adaptation by prebiotics and probiotics (54). The TY-11 might be used to lessen the amount of lactose in the intestine of lactose-intolerant people.

According to predictions, the TY-11 genome was unable to synthesize the majority of the 20 common amino acids. Some amino acids’ incomplete biosynthesis pathways were present in this genome. Although some research suggested that subsp. *indicus* may also be found in some nutrient-rich settings, such as yogurts (5), the genome of the strain TY-11 reflects the environment that it can live in the gut, where it may absorb peptides and amino acids from its surrounding environment.

### Comparative genomic study

#### The TY-11 entire genome is represented by *Lactobacillus delbrueckii* core genome, TY-11 non-core genome, and subsp. *indicus* core genome

Since we computed the core genome using a 95% threshold, our definition of the “core” genome did not require that a gene be missing in 100% of other strains of subspecies *indicus* (four genomes). For example, the TY-11 non-core genome had galactose-6-phosphate isomerase subunit LacB (LPJCKDFM_00598), and galactose-6-phosphate isomerase subunit LacA (LPJCKDFM_00599), which were variable in subspecies *indicus* (6). In enzymology, galactose-6-phosphate isomerase (lacA, lacB; EC5.3.1.26 = K01819) is the enzyme that catalyzes D-galactose 6-phosphate to D-tagatose 6-phosphate. This enzyme plays a role in the metabolism of galactose (53). Another gene of the non-core genome was sucrose phosphorylase encoded by LPJCKDFM_01520–01522. The enzyme sucrose phosphorylase (K00690, EC 2.4.1.7) is crucial for the regulation of several metabolic intermediates and the breakdown of sucrose. The transformation of sucrose to D-fructose and -D-glucose-1-phosphate is catalyzed by it (11). Although the fermentation of sucrose is more variable in subspecies *indicus*, the TY-11 bore these genes (4).

On the other hand, out of 17 distinct genes of the TY-11 genome, two genes, alpha, alpha-phosphotrehalase (LPJCKDFM_00870), and trehalose operon repressor (LPJCKDFM_00871), were related to trehalose metabolism and regulation, which were missed in other genomes of subspecies *indicus* (4, 6). Previously, Dellaglio et al. (4) and Germond et al. (3) distinguished subspecies *indicus* from subspecies *lactis* based on the trehalose and maltose metabolism (3, 4). The genes for trehalose metabolism and control were present in our strain, despite the fact that it belonged to the subspecies *indicus*. However, no genes related to maltose metabolism were identified.

#### Comparisons between subsp. *indicus* and *bulguricus* core genomes

In the category of “solely *bulgaricus* but not as *indicus* core genes,” out of 10, 2 genes were deficient in the strain TY-11, such as phosphate acetyltransferase (pta) and 50S ribosomal protein L36 (rpmJ). Phosphate acetyltransferase (pta) is an enzyme of the AckA-Pta pathway. This pathway, which is composed of two enzymes, produces acetate in both anaerobic and aerobic conditions. Phosphoenzyme A (Pta) produces acetyl-phosphate from acetyl-coenzyme A, while Acetate Kinase (AckA) produces acetate from acetyl-phosphate in a reaction connected to the synthesis of ATP. The TY-11 lacked Pta but had AckA in the AckA-Pta pathway. Principally, The TY-11 was primarily a strictly homofermentative anaerobic bacteria that could only undergo homolactic fermentation and did not require maintaining acetate for its metabolism and ATP. It was characteristic of most lactic acid bacteria and suited to the gut’s anaerobic environment (17) (discussed in Section Central Carbohydrate Metabolism). Another protein, ribosomal L36 is the smallest protein of the large subunit (50S) of the bacterial ribosome. It has a specific and key role in the organization of the 23 S rRNA. Corina et al. (55) speculated that Archaea and Eucarya have used other means to achieve the same degree of stabilization (55). The TY-11 had a dearth of protein L36 and might be compensated with other alternative proteins from 33 large subunit ribosomal proteins encoded in its genome for stabilization and folding of 50S (Table S1). However, this strain had 8 of the 10 core genes, of which 2 were determined to be significant. Membrane protein insertase YidC (LPJCKDFM_00904, LPJCKDFM_01385 = K03217) encoding gene was one of them (discussed in Section Secretion System). The other core gene of this sort was Fe-S cluster assembly protein SufB (LPJCKDFM_01855). This is a fundamental cofactor in proteins engaged in several common cellular functions, including respiration, central metabolism, ribosome synthesis, DNA replication, and repair (56). According to our findings, the TY-11 genome exhibited similarity with the strains of subspecies *bulgaricus* due to close ANI value. For example, the TY-11 draft genome showed 97.24% ANI with the nearest genome sequence of *bulgaricus* (*Lactobacillus delbrueckii* subsp. *bulgaricus* JCM 1002, GCA_000056065.1).

On the contrary, 37 core genes were annotated only for “at least 95% *indicus* but not for core *bulgaricus.”* The TY-11 genome carried all 37 genes for its function. The draft genome of the TY-11 manifested 98.980% ANI with the nearest genome of subspecies *indicus* (*Lactobacillus delbrueckii* subsp. *indicus* JCM 15610, GCA_001908415.1). Because of the close ANI value, the TY-11 genome demonstrated similarity with the strains of subspecies *indicus*. To take an example, a two-component system activity regulator YycH (LPJCKDFM_01012) encoding gene was a crucial gene found in this category. YycH regulates the activity of the essential YycFG two-component system. Cell wall homeostasis is regulated by the YycF-YycG system, suggesting that either an increase or decrease in YycF activity can have an impact on this homeostatic process (57). Another gene of this group was co-chaperone GroES (LPJCKDFM_00449). In total, these 37 genes reflected model probiotic characteristics of subsp. *indicus* (including the TY-11 genome).

Overall, the TY-11 genome contained all the core genes (95% threshold) of subspecies *bulgaricus* and *indicus,* except two genes: phosphate acetyltransferase (pta) and 50S ribosomal protein L36 (rpmJ). These two genes were not essential for the strain TY-11. The TY-11 genome had 37 core genes of the subsp. *indicus*, which were absent in the core genome of the most widely used subsp. *bulgaricus*.

### Phenotypic tests

The purpose of the phenotypic tests was to confirm the relevant genotypes of the strain TY-11. A key selection criterion for novel and effective probiotics is antimicrobial activity. The isolate TY-11 showed a strong anti-*Escherichia coli* ATCC 8739 activity. The result of this study is approximately similar to the WDS-7 strain that Changjun et al. (5) tested with (5). The anti-*E. coli* activity in our investigation was most likely caused by the organic acid, specifically lactic acid (pH 4.22 ± 0.19). The isolate TY-11’s antibacterial action was found to have a genetic basis for certain compounds, including organic acids, hydrogen peroxide, bacteriocins, and others as explained in Section Characterization of Antimicrobial Activity from Genomic Study and Mining Bacterial Genomic DNA for Bacteriocins. More specific *in vitro* laboratory testing is required to explore the particular antimicrobial action of the compounds released by this isolate. This involves testing the pathogens against the TY-11 culture broth supernatant with neutral pH and the broth supernatant treated with trypsin and catalase.

According to the antibiotic sensitivity test, the strain TY-11 was sensitive to all tested antibiotics. Changjun et al. (5) used the WDS-7 strain, a subsp. *indicus* strain, to perform an antibiotic sensitivity test. The WDS-7 strain exhibited resistance against vancomycin (30 µg/100 µL), gentamicin (10 µg/100 µL), kanamycin (30 µg/100 µL), streptomycin (10 µg/100 µL), and metronidazole (5 µg/100 µL) (6). Since a probiotic should be susceptible to antibiotics, strain TY-11 performed better than strain WDS-7. No antibiotic-resistant gene was found in the TY-11 genome by the Comprehensive Antibiotic Resistance Database (CARD) (explained in Section Genome Composition Analysis of the Genome TY-11 by Assembly and Annotation). Thus, the phenotype and genotype together confirmed that this strain was the suitable probiotic in terms of antibiotic sensitivity.

### Conclusions

In our current investigation, we employed bioinformatics analysis and comparisons to other probiotic genomes of *Lactobacillus delbrueckii*, which revealed a number of common and unique excellences of the probiotic *Lactobacillus delbrueckii* subsp. *indicus* TY-11 that are likely to be important to illustrate its intestinal residence and probiotic roles. Remarkably, 2 of the 17 distinct genes that were identified only in the TY-11 genome but not in the other genomes of the subspecies *indicus* were connected to trehalose metabolism and regulation. When we compared the core genes among the strains of subspecies *bulgaricus* and *indicus*, we found that the TY-11 genome overall had all the core genes (95% threshold) of the subspecies *bulgaricus* and *indicus,* except two genes—phosphate acetyltransferase (pta) and 50S ribosomal protein L36 (rpmJ). The strain TY-11 did not require these two genes for its cellular function. This strain also possessed no genes related to harmful substances or antibiotic resistance, which is another probiotic trait.

Besides genomic studies, some phenotypic characteristics of the strain TY-11, including antibacterial activity, antibiotic sensitivity, and lactose fermentation, were unveiled by *in vitro* laboratory tests in this study. The potential probiotic TY-11 had a strong anti-*E*. *coli* activity. It can be a cost-effective option for the treatment of human illness caused by diarrhea and other food-related infections linked to pathogenic *E. coli*.

## MATERIALS AND METHODS

### Isolation of bacterial samples

#### Isolation of bacterial samples based on morphology, acid secretion efficiency, and anti-*E*. *coli* activity

Probiotic species were isolated from a number of samples of yogurts in Tongi, Gazipur, Bangladesh. 1 mL of 10^−6^ decimal diluted sample [suspended in normal 0.9% (wt/vol) saline solution] was spread on 20–25 mL MRS (de Man Rogosa Sharpe, Oxoid, UK) agar medium, and at 37°C the Petri-dishes were incubated for 24–72 h. Based on morphology, several bacterial colonies showed similarity with *L. delbrueckii* subsp. *indicus* were selected from MRS agar media. To purify colonies, the isolates were streaked on the same media and finally, the pure colonies were transferred to MRS broth with 15% glycerol for further research.

We also observed the acid production capacity of the isolates in MRS broth after 24 h of incubation at 35°C. The anti-*E. coli* activity of the isolates was examined in a Tryptic Soy Agar (TSA) medium. 100 µL supernatant of the cell suspension broth of the isolates (24 h of incubation at 35˚C) was placed in a 7 mm diameter well in TSA media having a lawn of *Escherichia coli* ATCC 8739. 100 µL of *E. coli* (10^5^ CFU/mL) was added as a pour plate technique in TSA media. We purchased *Escherichia coli* ATCC 8739 from Microbiologics, Inc., USA, by an authorized distributor, Bangladesh Labware Corporation, Dhaka, Bangladesh. We preferred three isolates for 16S sequencing based on morphology, acid secretion efficiency, and anti-*E*. *coli* activity.

#### 16S sequencing and molecular identification of bacterial samples

Following the product’s instructions, the MRS-agar grown cultures of three isolates (TY-11, TB-3, and TY-3) were put to use for the phenol-chloroform chemical lysis procedure to isolate genomic DNA. The quantity and purification of the DNA were assessed using a Nanodrop spectrophotometer ND2000 (Thermo Scientific, USA) following the extraction process for DNA. The pureness of the DNA was determined by looking at the 260/280 ratio, or absorbance at 260 and 280 nm. For pure DNA, the ratio is more than 1.8. In accordance with the technique previously reported by Rahman et al. (2017) (15), 16S rDNA amplification and sequencing were carried out in this work. The following were the standard 16S rRNA primers sequence for PCR (polymerase chain reaction) amplification: 1492r (5′- GGTTACCTTGTTACGACTT-3′) and 27 f (5′-AGAGTTTGATCCTGGCTCAG-3′).

#### Colony morphology of the isolate TY-11

We examined the colony morphology of the isolate TY-11 under The Leica MZ9.5 (Germany), a powerful stereo microscope with an excellent 9.5:1 zooming ratio including magnification levels up to 480×.

#### Bacterial samples analysis under transmission electron microscope

The TY-11 cells were cultured in MRS broth overnight. The overnight grown cultures were centrifuged with 500 RPM at 4° C. After centrifugation, we used 10 µL of distilled water to suspend the pellet. The suspended bacterial samples were applied to the grid, and it was incubated for 1 minute. We used a plasma cleaner (power: low, 12 s) to hydrophilize the grid prior to use. Next, 1.8% uranyl acetate solution was used to stain the samples, and they were left to incubate for one minute. A VELETA CCD Camera (Olympus Soft Imaging Solutions) placed on a JEM 1010 transmission electron microscope (JEOL) was used to capture images of the bacterial samples on the grid. A total of three biological samples were prepared for these experiments and 10 cells were measured using the microscope’s default scale.

### Genome sequencing and QC check of genome sequence

The TY-11 cells were cultured in MRS broth overnight. The overnight grown cultures were centrifuged with 500 RPM at 4° C. The genomic DNA extraction process was described in Section 16S Sequencing and Molecular Identification of Bacterial Samples. Illumina DNA Prep was used to prepare the DNA library of the isolate TY-11. To tagment DNA, this technique, utilizing Bead-Linked Transposomes (BLT), breaks and tags DNA using adapter sequences. Prior to PCR amplification, the adapter-tagged DNA was cleaned on the BLT during the post-tagmentation step. A limited-cycle PCR technique was then used to amplify the tagmented DNA. Index 1 (i7) and Index 2 (i5) adapters, as well as the sequences needed for sequencing cluster creation, were added during the PCR stage. To purify the amplified library, a double-sided bead purification technique was applied. The genome of TY-11 was sequenced using a whole-genome (WGS) strategy (30-fold genome coverage) with NextSeq 550 (Illumina, USA).

The raw data of the genome sequence of the isolated TY-11 were checked by FastQC (version 0.11.9) and fastp (version 0.19.7, https://github.com/OpenGene/fastp).

### Genome composition analysis by assembly and annotation

The genome data were assembled using Unicycler 0.5.0 (58) and SPAdes 3.15.4 (59). Annotation was conducted by PROKKA (version 1.13) (18). A high-throughput prophage sequence annotation, Phigaro was used for searching prophages in the isolate TY-11 (7). The Resistance Gene Identifier (RGI) (Version 1.1.1) of the Comprehensive Antibiotic Resistance Database (CARD) was utilized to predict antibiotic resistance genes (60).

### Phylogenetic study and identification

The new genome was compared with 261 RefSeq genomes of *Lactobacillus delbrueckii*. All single-copy genes that were contained in the new genome and also present in at least 95% of the total 262 genomes (261 RefSeq genomes + TY-11genome) were selected. These were turned out as 332 genes. The sequences of amino acids of all these genes were extracted and saved into separate files-one file per gene. All amino-acid sequences were aligned in a single file.

Then, nucleotide sequences were re-extracted separately from 332 amino acid sequences. All nucleotide sequences were aligned in a single file. Combined nucleotide sequence alignment for 332 genes from 262 genomes was used for phylogeny. The Neighbor-Joining method was deployed to create a phylogenetic tree (61) implemented in MEGA 11 (62).

As a core genome, 353 genes are present in all 5 *L*. *delbrueckii* subsp. *indicus* genomes selected from the first tree (TY-11 genome and 4 subsp. *indicus* genomes). The amino-acid sequence alignment was run for those 353 genes (separately for each gene). The Neighbor-Joining approach was employed to infer the second tree employing five genomes of *L. delbrueckii* subsp. *indicus* (based on aligned amino-acid sequences of 353 genes) (61) To evaluate tree accuracy, the bootstrap approach of 1,000 bootstrap repeats was utilized. Evolutionary analyses were conducted in MEGA 11 (62).

Identification of the isolated strain TY-11 was conducted by the 16S sequence from the draft genome. The 16S sequence identity was searched by an online search tool, Nucleotide BLAST Nucleotide BLAST (https://blast.ncbi.nlm.nih.gov/). We searched Average nucleotide identity (ANI) with the genome sequence by Python package pyani (63).

Sequence similarity was checked with aligned 353 core gene sequences by Similarity Matrix: MATCH in BioEdit (64). In addition, subspecies-specific genes were searched in the genomes for subspecies-level identification.

The tests for fermentation of lactose were conducted using BioMérieux’s API biochemical kit and its responsible genes were identified. Gram staining test was conducted using the Coica (2005) method (65).

### Functionality analysis of genome by KEGG annotations

Kyoto Encyclopedia of Genes and Genomes (KEGG) online annotations were used for protein-coding genes of the genome TY-11, using the Blastkola website (https://www.kegg.jp/blastkoala). On the Blastkola website, query amino acid sequences of the TY-11 genome in FASTA format annotated from PROKKA were submitted. KO (K number) assignment was searched using SSEARCH computation by KEGG’s internal annotation tool in Prokaryotes from the KEGG GENES database. Each gene’s biological pathway was obtained through KEGG orthology (https://www.kegg.jp/kegg/pathway.html) (66).

### Mining bacterial genomic DNA for bacteriocins

BAGEL database enables researchers to mine bacterial (meta-) genomic DNA for bacteriocins and RiPPs. In addition, they offer databases as well as a BLAST against the primary peptide databases. The query gene sequences of the TY-11 genome in FASTA format annotated from PROKKA were submitted to the BAGEL database (http://bagel4.molgenrug.nl/). Genes and proteins for synthesizing bacteriocins were annotated with the BAGEL database. Core-peptide database searches and/or HMM motif searches across related context genes were used to identify gene clusters of interest. The mining database was linked to UniProt and NCBI as well as references to previous research (67).

### Genome comparisons (TY-11 entire genome, *Lactobacillus delbrueckii* core and TY-11 non-core genome, and subsp. indicus core genome)

We computed the core genome using a 95% threshold. We compared all single-copy genes that were obviously contained in the draft entire genome of the isolate TY-11 and also present in at least 95% of the total 261 RefSeq genomes of *Lactobacillus delbrueckii*. Second, we compared all single-copy genes that were obviously contained in the draft entire genome of the isolate TY-11 and also present at least 95% of the total 4 RefSeq genomes of subsp. *indicus*. KEGG online annotations were used to search for the TY-11 non-core genes that were absent at least 95% of the total 4 RefSeq genomes of subsp. *indicus*. KO (K number) assignment was searched using SSEARCH computation by KEGG’s internal annotation tool in Prokaryotes from the KEGG GENES database. Each gene’s biological pathway was obtained through KEGG orthology (https://www.kegg.jp/kegg/pathway.html) (66).

To compute the core genome of subsp. *bulgaricus* and *indicus*, we compared all single-copy genes of 69 RefSeq genomes in subsp. *bulgaricus,* and all single-copy genes of 5 RefSeq genomes in subsp. *indicus*.

### The phenotype tests of the isolate TY-11

For the antimicrobial activity test of the isolate TY-11, with a few changes, we used the straightforward spot-on lawn agar method. TSA (Tryptic Soy Agar) medium was used to assess the antagonistic effects on *Escherichia coli* ATCC 8739 (68). On TSA media with a lawn of pathogenic microorganisms, 50 µL supernatant of the isolate TY-11 cell suspension broth was added (24 h of incubation at 35°C).

The anti-*E. coli* activity of the isolate TY-11 was retested by the agar-well diffusion method of Chidre et al. (69) with a few changes (69). To provide a comparison, we grew the isolate TY-11 and the control separately in MRS broth for 24 h at 35°C. In TSA media, the antagonistic action against the pathogen was evaluated. A 7-mm-diameter well in TSA media, surrounded by a lawn of *Escherichia coli* ATCC 8739 (100 µL at a concentration of 10^5^ CFU/mL), was filled with 100 µL cell-free supernatant of the isolate TY-11 culture broth and the control. As a control, *Lactobacillus acidophilus* (LA1063) was used and isolated from lyophilized powder purchased from Synbiotech (Taiwan). The strain was identified at the species level from our end by 16S sequencing. There were two tests conducted to determine the antimicrobial activity by agar well diffusion method. Strong, intermediate, and mild inhibitions were evaluated for inhibition zones exceeding 20 mm, 10–20 mm, and less than 10 mm, respectively (69).

The probiotic isolate TY-11 was subjected to an antibiotic sensitivity test using a modified version of Chetan et al.’s (70) methodology. 8 mL of MRS agar (24.66 ± 4.16×107 CFU/mL of the isolate TY-11) was over-adjusted on MRS agar that had already solidified (70). 100 µL of ampicillin (10 µg/100 µL), vancomycin (30 µg/100 µL), gentamicin (10 µg/100 µL), kanamycin (30 µg/100 µL), streptomycin (10 µg/100 µL), erythromycin (15 µg/100 µL), tetracycline (30 µg/100 µL), chloramphenicol (30 µg/100 µL), tylosin (30 µg/100 µL), and clindamycin (10 µg/100 µL) were added in a 7-mm diameter well in MRS agar media with an upper layer of the probiotic bacterial strain. At 37°C, the test Petri dishes were incubated for a full day. As a control, *Lactobacillus acidophilus* (LA1063) was used. There were three tests conducted to determine the sensitivity of each antibiotic.

## Data Availability

The genomes generated and analyzed during the current study are available at NCBI under Project PRJNA975465. The associated Sequence Read Archive (SRA) and BioSample accession numbers are SRR24696316 and SAMN35326563, respectively.

## SUPPLEMENTAL MATERIAL

Fig. S1 spectrum.03470-23-s0001.xlsx Phylogenetic tree-1.

Table S1 spectrum.03470-23-s0002.xlsx Genome sequence.

Table S2 spectrum.03470-23-s0003.xlsx Prophages in the TY-11 genome.

Table S3 spectrum.03470-23-s0004.xlsx Microorganisms in the phylogeny.

Table S4 spectrum.03470-23-s0005.xlsx ANI crosstable for the strains of six subspecies.

Table S5 spectrum.03470-23-s0006.xlsx Central carbohydrate metabolism.

Table S6 spectrum.03470-23-s0007.xlsx Folate and coenzyme: a biosynthesis pathway.

Tables S7 and S8 spectrum.03470-23-s0008.xlsx ABC transporters and phosphotransferase system.

Tables S9 to S14 spectrum.03470-23-s0009.xlsx Tables S9 to S14.

ASM does not own the copyrights to Supplemental Material that may be linked to, or accessed through, an article. The authors have granted ASM a non-exclusive, world-wide license to publish the Supplemental Material files. Please contact the corresponding author directly for reuse.
